# The Lag Effects and Vulnerabilities of Temperature Effects on Cardiovascular Disease Mortality in a Subtropical Climate Zone in China 

**DOI:** 10.3390/ijerph110403982

**Published:** 2014-04-11

**Authors:** Jixia Huang, Jinfeng Wang, Weiwei Yu

**Affiliations:** 1 State Key Laboratory of Resources and Environmental Information System, Institute of Geographic Science and Natural Resource Research, Chinese Academy of Sciences, Beijing 100101, China; E-Mail: huangjx@lreis.ac.cn; 2 Key Laboratory of Surveillance and Early Warning on Infectious Disease, Chinese Center for Disease Control and Prevention, Beijing 102206, China; 3 School of Public Health and Social Work, Institute of Health and Biomedical Innovation, Queensland University of Technology, Brisbane 4059, Australia

**Keywords:** extreme temperature, cardiovascular disease, heat-related, cold-related, lag effect

## Abstract

This research quantifies the lag effects and vulnerabilities of temperature effects on cardiovascular disease in Changsha—a subtropical climate zone of China. A Poisson regression model within a distributed lag nonlinear models framework was used to examine the lag effects of cold- and heat-related CVD mortality. The lag effect for heat-related CVD mortality was just 0–3 days. In contrast, we observed a statistically significant association with 10–25 lag days for cold-related CVD mortality. Low temperatures with 0–2 lag days increased the mortality risk for those ≥65 years and females. For all ages, the cumulative effects of cold-related CVD mortality was 6.6% (95% CI: 5.2%–8.2%) for 30 lag days while that of heat-related CVD mortality was 4.9% (95% CI: 2.0%–7.9%) for 3 lag days. We found that in Changsha city, the lag effect of hot temperatures is short while the lag effect of cold temperatures is long. Females and older people were more sensitive to extreme hot and cold temperatures than males and younger people.

## 1. Introduction

Extreme temperatures increase cardiovascular disease (CVD) mortality [1,2,3,4,5]. Global climate changes have increased the frequencies of cold snaps and heat waves [6,7]. To some extent, this may have an impact on cardiovascular disease mortality. The association curve between temperature and CVD mortality is U-, V-, or J-shaped [8,9]. Generally, there are a cold temperature threshold and a hot temperature threshold of the U-shaped curve. For the U-shaped curve, CVD mortality increases as temperatures fall below the cold temperature threshold and also as temperatures increase above the hot temperature threshold. From previous studies, we also know that association curves differ and vary with latitude [10,11].

Extreme temperatures have a lag effect on CVD mortality [12,13]. Both the extreme temperature of that current day and that of previous days affect the CVD mortality. Previous research has shown that heat- and cold-related CVD mortality have different lag periods and also that the impacts of temperature on CVD mortality change on different lag days [13,14,15,16,17]. Associations between temperature and mortality also differ with age and gender. Extreme temperatures affect more older people than younger ones [18], and females have been shown to be more sensitive than males to extreme temperatures [19,20,21].

To date, studies of the associations between temperature and CVD mortality have been conducted in mostly Europe and the United States [6,8,9,13]. In China, some studies have occurred in Beijing and Tianjin [11,19,22], but research on the Yangtze River basin, located in the subtropical region of China, has been lacking. In this study, this association is studied in Changsha City, one of the most important cities of the Yangtze River basin. We mainly focused on three questions: (1) Do temperature thresholds exist in Changsha? (2) What is the lag effect of temperature-related CVD mortality here? (3) How does temperature affect CVD mortality in people of different ages and gender?

## 2. Material and Methods

### 2.1. Study Area

**Figure 1:**
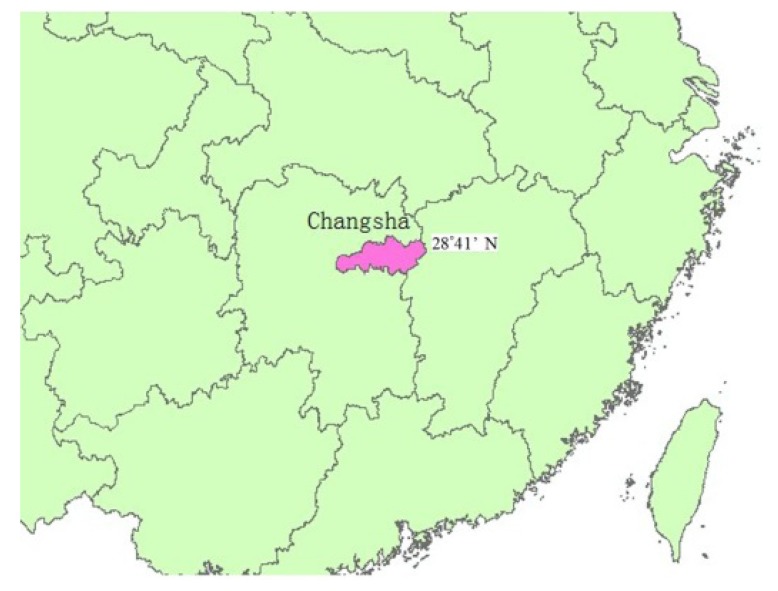
Geographical Location of Changsha in China.

Our research area was Changsha City (27^°^51′–28^°^41′ North, 111^°^52′–114^°^15′ East), the capital of Hunan Province located in the middle reaches of the Yangtze River basin (Figure 1). According to the 2011 census, Changsha covers 11,816.0 km^2^ and has a population of 6.57 million [23]. This area has a typical subtropical climate with high temperatures in the summer (Jun–Aug) and cold temperatures in the winter (December–February).

### 2.2. Mortality Data

We obtained CVD mortality data of Changsha City between the dates of 1 January 2008 and 31 December 2011 (1,461 days) for all ages from the Chinese Centers for Disease Control and Prevention. The causes of CVD were classified according to the International Classification of Disease 10th version (ICD-10:I00-I79). We calculated daily CVD death counts for all ages, those aged ≥65 years, those aged <65 years, males, and females. 

### 2.3. Meteorological and Air Pollution Data

Meteorological data was obtained from the China Meteorological Data Sharing Service System. There are two meteorological monitor stations in Changsha City. We averaged the meteorological data from both stations, including daily mean temperature, daily maximum temperature, daily minimum temperature, daily mean barometric pressure, and daily mean relative humidity. We used Pearson’s coefficient to calculate the correlations between daily mean temperature and daily maximum and daily minimum temperatures of 0.97 and 0.98, respectively. Because detailed air pollution data (such as PM_10_, PM_2.5_, SO_2_, and NO) were not available, the air pollution index (API) [24] from the Changsha Environmental Protection Agency was used instead. Based on World Health Organization and European Centre for Environment and Health methodology, air pollution indices (APIs) were calculated using respirable particulate matter (PM_10_), sulfur dioxide (SO_2_), and nitrogen dioxide (NO_2_) measurements obtained by the monitoring stations [25].

### 2.4. Statistical Analyses

The generalized additive model (GAM) [26] has been the standard reference method for analyzing associations between environmental factors and mortality with time series data [27,28]. Akaike information criterion (AIC) [29] was used to test the fitting degree of the time series data. GAMs were performed using R, version 2.14.0, with the “mgcv” package [30]. The “dlnm” package in R was also used to generate distributed lag nonlinear models (DLNM) [31]. The association between temperature and CVD mortality was analyzed by two steps.

First, the association curve between temperature and CVD mortality was plotted to determine whether the temperature thresholds existed. We used mean, minimum, and maximum temperatures as temperature indicators. The fitting degrees of these indicators showed that mean temperature had the best estimation of temperature-related CVD mortality [32]. The distributed lag effects of confounding variables (*i.e*., air pollution index, barometric pressure, relative humidity, and wind speed) were considered using the GAM. For each confounding variable, we examined the delayed effect at 0–7 lag days. The results showed that the AIC value of the GAM was lowest when all confounding variables, except wind speed, had seven lag days. 

We used the GAM, as follows, to detect the temperature thresholds [17,33]:

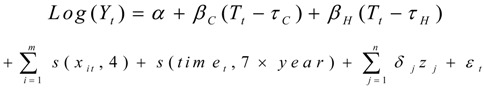
(1)
where t denotes the day of observation; *Y_t_* represents the daily death count on day t; *T_t_* means the daily mean temperature; *τ_C_* and *τ_H_* refer to the cold and heat temperature thresholds, respectively; (.)denotes the smoothing splines function; *x_it_* denotes the covariates such as API, barometric pressure, and relative humidity; *tim e_t_* refers to the calendar time; *z_j_* denotes factors such as week day and holidays; *α* denotes intercept term; *β_C_*, *β_H_* and *δ_j_* denote the coefficient; and *ε_t_* is the residual. The long-term and seasonal trends were controlled by smoothing calendar time with seven degrees of freedom/year, according to previous studies [28]. Four degrees of freedom/year were used to smooth API, barometric pressure, and relative humidity [13].

The minimum AIC value was used to detect the temperature thresholds [11,17]. AIC values were calculated iteratively for model (1) using increasing increments of 1 °C for *τ_C_* (0 °C–35 °C) and decreasing increments of 1 °C for *τ_H_* (35 °C–0 °C). In this iterative process, *τ_H_* should be greater than *τ_C_*. This method has been widely used in previous studies [11,17]. We adjusted the threshold within the DLNM framework for up to 30 lag days for mean temperature. For other confounding variables such as API, barometric pressure, and relative humidity, we computed the AIC values for up to 30 lag days. When the lag period was seven days, the AIC value was smallest. So in this research, we chose the lag of 0–7 days for the confounders.

In the second step, we examined the distributed lag relationships of cold- and heat-related CVD mortality lagged up to 30 days. We used DLNM to solve the collinearity between adjacent lag days [31]. We analyzed cold- and heat-related CVD mortality for all ages, those aged ≥65 years, those aged <65 years, males, and female.

### 2.5. Sensitivity Analyses

We used various smooth degrees of freedom to analyze long-term trend sensitivity (3–10 df) and meteorological data sensitivity (3–7 df). No substantial changes were observed in these analyses.

## 3. Results

**Figure 2:**
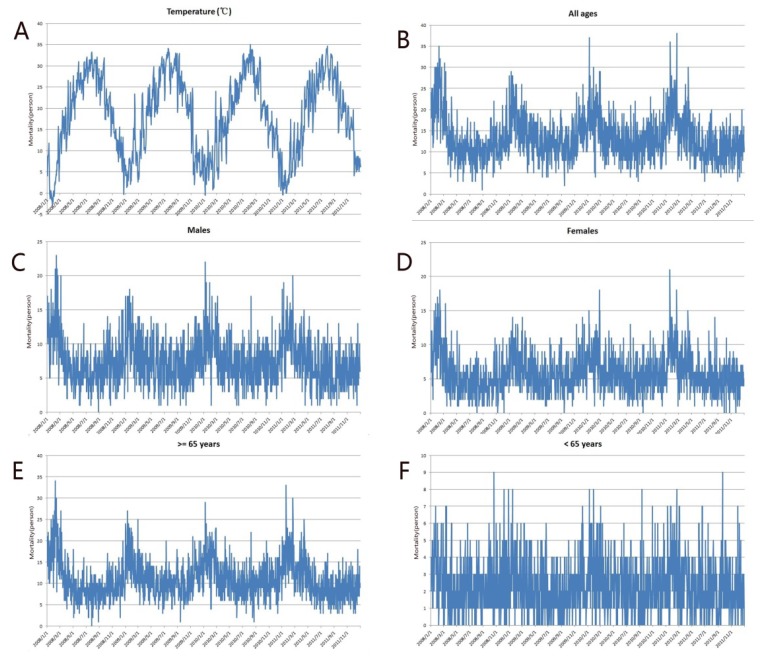
Time series of daily mean temperature (**A**), and time series of CVD mortality for (**B**) all ages, (**C**) males, (**D**) females, (**E**) those ≥65 years, (**F**) those <65 years, all of the vertical axis in these subfigures denote death count.

There were a total of 19,418 CVD deaths between January 2008 and December 2011 with 82.5% of these deaths occurring in those ≥65 years. The death counts for males and females were 10,912 and 8,506, respectively. CVD mortality followed a strong seasonal pattern with peaks in the winter and troughs in the summer for all groups except those <65 years (Figure 2). The daily mean temperature and daily barometric pressure also fluctuated with the seasons, peaking in the summer and reaching a trough in the winter (Figure 2F).

The daily mean CVD death counts for all ages and those aged ≥65 years were 13.3 and 11, respectively. Daily mean, minimum, and maximum temperatures were 18.3 °C, 15 °C, and 22.6 °C, respectively. Table 1 describes the distribution of these variables.

**Table 1:** The Distribution of CVD Mortality and Meteorological Data in Changsha.

			Percentile			
	Mean(SD)	Minimum	25%	50%	75%	Maximum
CVD All ages	13.3	1	10	12	16	38
CVD 65+	11.0	0	8	10	13	34
CVD 65−	2.3	0	1	2	3	9
CVD Male	7.5	0	5	7	10	23
CVD Female	5.8	0	4	5	7	21
API	68.7	11	53	67	82	443
AT	18.3	−2.8	10.1	19.2	26.4	35
MaxTemp	22.6	−2.1	14.9	23.4	31.2	40.7
MinTemp	15.0	−5.3	7.2	16.3	23	30.7
RH	74.8	29	66	76	85	97
Wind	2.0	0.4	1.4	1.9	2.5	5.7

Note: CVD 65+: CVD mortality for aged ≥ 65 years; CVD 65−: CVD mortality for aged < 65 years; API: air pollution index; AT: daily average temperature; MaxTemp: daily maximum temperature; MinTemp: daily minimum temperature; RH: daily relative humidity.

**Figure 3:**
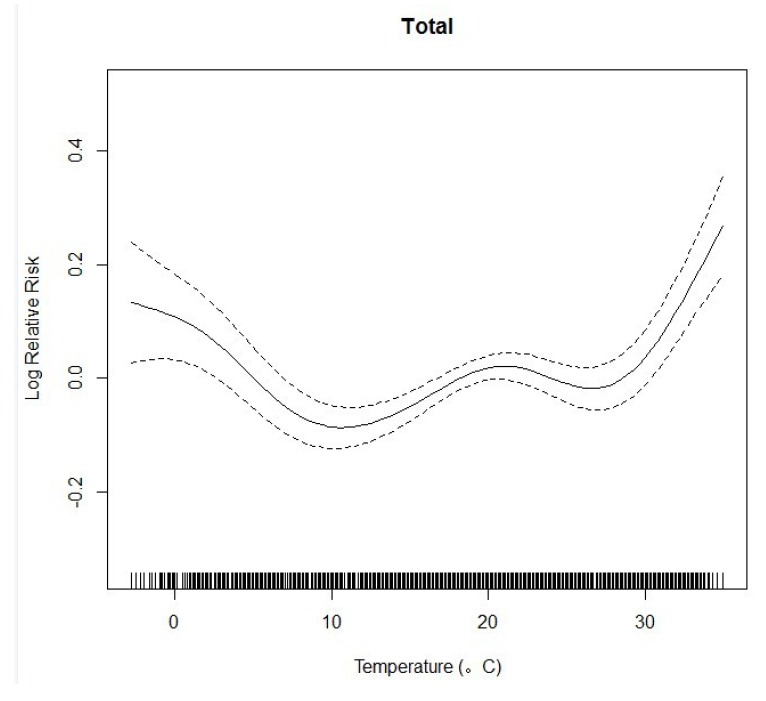
The Association Curve between Log Relative Risk and Temperature.

We plotted the relationship curve between the log relative risk and the mean temperature of the current day for all ages. This resulted in an U-shaped association curve (Figure 3). 

We identified two temperature thresholds: hot (29 °C) and cold (10 °C). When temperatures were below 10 °C or above 29 °C, the relative risk for CVD mortality increased approximately linearly along with decreases (and increases) in temperature. The risk curve is relatively flat when the temperature is between 10 °C and 29 °C.

**Figure 4:**
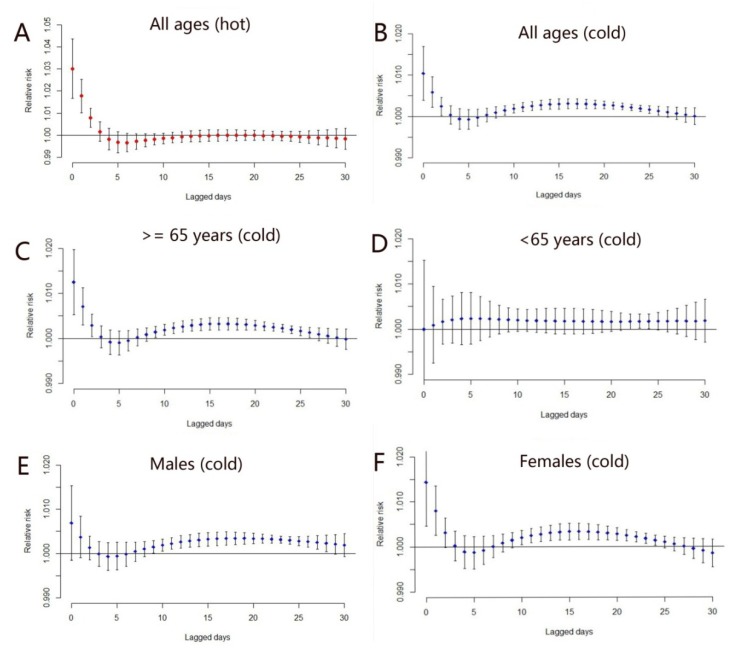
The distributed lag period of heat-related mortality for all ages (**A**); The distributed lag period of cold-related mortality for (**B**) all ages, (**C**) those ≥65 years, (**D**) those <65 years, (**E**) males, and (**F**) females.

The lag period of heat-related mortality was only 1–3 days. Figure 4A displays the lag effect of heat-related mortality for all ages. We found that CVD mortality was affected by hot temperatures during the current day and in the previous two days. The lag period of heat-related mortality was also present in the other groups (those ≥65 years, those <65 years, males, and females). In these groups, the lag periods possessed the same characteristics as that of all ages (not shown for the approximate same shapes).

**Table 2:** The Cumulative Effect of Cold- (25 lag days) and Heat- (3 lag days) Related CVD Mortality.

	Cold Effect (<10 °C)		Heat Effect (>29 °C)	
	Estimate (%)	95%CI	Estimate (%)	95% CI
All ages	6.6 *	5.2–8.2	4.9 *	2.0–7.9
Older age (≥65)	7.2 *	5.3–9.1	4.4 *	1.3–7.7
Younger age (<65)	5.4 *	2.9–8.1	5.2	−0.1–11.4
Males	6.4 *	5.6–8.2	3.9 *	0.1–7.9
Females	6.7 *	4.6–8.8	6.0 *	2.0–10.9

Notes: * *p* < 0.05. 95% CI: 95% confidence interval

The lag period of cold-related mortality varied between the different groups (Figure 4B–F). Cold temperatures in the previous two days affected all ages, those ≥65 years, and females. However, they did not increase the death risk of those <65 years and of males. Cold temperature in the previous 10–25 days affected all groups except those <65 years. 

Because of the different lag periods for the cold- and heat-related mortality (Figure 4), we computed the cumulative effects of 25 and three lag days for cold- and heat-related mortality, respectively (Table 2). Cold temperatures affected all groups. A 1 °C temperature decrease below the cold temperature threshold (10 °C) was associated with an overall increase (25 days) in CVD mortality of 6.6% (95% CI: 5.2%–8.2%) for all ages, with 7.2% (95% CI: 5.3%–9.1%) and 5.4% (95% CI: 2.9%–8.1%) for the elderly and the younger. The lag effect of hot temperatures did not affect those <65 years. A 1 °C increase in temperature above the hot temperature threshold (29 °C) was associated with overall increase (three days) in CVD mortality of 4.9% (95% CI: 2.0%–7.9%), with 3.9% (95% CI: 0.1%–7.9%) for males and 6.0% (95% CI: 2.0%–10.9%) for females.

## 4. Discussions

Temperature-related mortality exhibits spatial heterogeneity according to geography [10]. To date, studies of temperature-related CVD mortality has been lacking for the Yangtze River basin. The Yangtze River basin has a subtropical climate with relatively high summer and low winter temperatures [34]. Some studies, mainly in northern parts of China, have been conducted [19,22].However, the results of these studies cannot be applied to the Yangtze River basin because of spatial heterogeneity [11,35]. The effects of temperature on CVD mortality lasted three days for heat and 25 days for cold in Changsha city. Females were more vulnerable to both heat and cold weather and the older people were more sensitive to cold temperatures. 

Low temperatures affect the body’s circulatory system. Blood supplied to the skin decreases when exposed to cold air, which results in an accumulation of blood in central organs, and the excess blood is then disposed of by removing salt and water. Some blood is removed by the kidneys as urine while some settles in general intercellular spaces [36].

High temperatures increase the density of the blood. In hot environments, blood vessels in the skin will expand in order to maintain body temperature, resulting in sweat expelling from the body. This decreases the salt and water in the body, increasing blood density and, consequently, its propensity for clotting [36].

We found that the association curve between temperature and CVD mortality in Changsha is U-shaped with a cold temperature threshold at 10 °C and a hot temperature threshold at 29 °C. This result is different from those of previous works in other parts of the world [11,17,37]. Chung *et al.* [11] found that the curve was V-shaped in Seoul and Tokyo and J-shaped in Beijing and Taipei with a temperature threshold of nearly 30 °C in all those cities. Liu *et al.* [37] also found that the association between temperature and CVD mortality in Beijing was V-shaped with a temperature threshold of 21.3 °C. In Brisbane, Australia, Yu *et al.* [17] also found a V-shaped curve with a temperature threshold of 24 °C. Our study further confirms the spatial heterogeneity of temperature-related CVD mortality in China. Unlike northern China or Australia, most houses in Changsha cannot be supplied with heating in the winter due to socioeconomic and energy limitations [38]. This may be why a cold temperature threshold exists in Changsha. When daily mean temperatures are below 10 °C or above 29 °C, the local government should enact measures to protect CVD patients from sudden death.

This study showed that the effect of hot temperatures had a short lag period of only 1–3 days while that of cold temperatures had a long lag period of 10–25 days. This is consistent with the previous studies [13]. High and low temperatures affect different cardiovascular diseases. Generally, low temperatures will trigger myocardial ischemia and acute myocardial infarction [39,40], while high temperatures will trigger the congestive heart failure [36]. High temperatures will directly lead to CVD death while cold temperatures only have indirect effects [13]. This may explain why the lag periods are different for hot and cold temperatures.

Several studies found two to five lag days for cold-related mortality [19,41]. The lag period (10–25 days) in our study is obviously much longer than those studies. The previous studies showed that socioeconomic levels affect the lag period of cold temperatures [8,14,18]. Changsha has a lower socioeconomic level than Beijing or cities in the United States. This may explain the longer lag period for cold-related mortality in Changsha city.

This study also found that females were more sensitive than males to heat-related CVD mortality. The cumulative effects of heat-related CVD mortality for females were more than 1.5-fold higher than those for males, with an increase of 1 °C above the hot temperature threshold (29 °C). However, the male mortality risk is approximately equivalent to the female mortality risk for cold-related CVD deaths. Schwartz [20] and Tian *et al.* [19] found that females were more sensitive to extreme hot and cold temperatures than males. This may be because females have a higher risk for arrhythmia, ischemia, and high blood pressure, all of which are more affected by extreme hot and cold temperatures [21]. Older people were more sensitive than younger people to extreme hot and cold temperatures. The cumulative effects of cold-related CVD mortality for those ≥65 years was more than 1.4 times higher than those for people younger than 65 years, with a decrease of 1 °C below the cold temperature threshold (10 °C). For those ≥65 years, heat temperatures in the previous 0–2 days affected their CVD mortality. In contrast, for those <65 years, heat temperatures did not have this effect. We know that extreme temperatures increase CVD deaths among the elderly [41]. There are some limitations to this research. Because air pollution data such as PM_10_, PM_2.5_, or O_3_ is hard to find for Changsha, we used an air pollution index instead which may have introduced biases. However, the major air pollution index in Changsha (more than 80%) is composed of PM_10_. Another issue is that we only examined Changsha. Consequently, the results of this study cannot be applied to northern China. In the future, the relationship between temperature and CVD mortality should be examined in multi-city studies.

## 5. Conclusions

The study found that the effect of cold temperatures had a long lag period while that of hot temperatures had a short one. Females and older people were more sensitive to extreme hot and cold temperatures than males and younger people. These findings on the association of temperature on CVD mortality have implications for Changsha policymakers and future scientific work.
